# Serum Iron:Ferritin Ratio Predicts Healthy Body Composition and Reduced Risk of Severe Fatty Liver in Young Adult Women

**DOI:** 10.3390/nu9080833

**Published:** 2017-08-04

**Authors:** Nindy Sabrina, Chyi-Huey Bai, Chun-Chao Chang, Yi-Wen Chien, Jiun-Rong Chen, Jung-Su Chang

**Affiliations:** 1 School of Nutrition and Health Sciences, College of Nutrition, Taipei Medical University, 250 Wu-Hsing Street, Taipei 110, Taiwan; sabrina.nindy@gmail.com (N.S.); ychien@tmu.edu.tw (Y.-W.C.); syunei@tmu.edu.tw (J.-R.C.); 2 Department of Public Health, College of Medicine, Taipei Medical University, 250 Wu-Hsing Street, Taipei 110, Taiwan; baich@tmu.edu.tw; 3 Division of Gastroenterology and Hepatology, Department of Internal Medicine, Taipei Medical University Hospital, 252 Wu-Hsing Street, Taipei 110, Taiwan; chunchao@tmu.edu.tw; 4 Division of Gastroenterology and Hepatology, Department of Internal Medicine, School of Medicine, College of Medicine, Taipei Medical University, Taipei 110, Taiwan; 5 Nutrition Research Center, Taipei Medical University Hospital, 252 Wu-Hsing Street, Taipei 110, Taiwan

**Keywords:** non-alcoholic fatty liver disease, serum iron, serum ferritin, body composition, dietary pattern

## Abstract

Dysregulated iron metabolism is associated with altered body composition and nonalcoholic fatty liver disease (NAFLD); however, mechanisms underlying this association remain undefined. We investigated this association in 117 women. Middle-aged women (≥45 years old (y)) were heavier and had lower serum iron, higher serum hepcidin, ferritin, and severe NAFLD incidence than young adult women (<45 y). Age-adjusted linear regression analysis revealed that young adult women with the highest serum iron:ferritin ratio (Tertile 3) had a 5.08-unit increased percentage of muscle mass [β = 5.08 (1.48–8.68), *p* < 0.001] and a 1.21-unit decreased percentage visceral fat mass [β = −1.21 (−2.03 to −0.39), *p* < 0.001] compared with those with the lowest serum iron:ferritin ratio (Tertile 1; reference). The iron:ferritin dietary pattern, characterized by high consumption of beef, lamb, dairy products, fruits, and whole grains, and low consumption of refined carbohydrates (rice, noodles, and bread and pastries), and deep- and stir-fried foods, predicted a 90% [odds ratio: 0.10, 95% confidence interval: 0.02–0.47, *p* < 0.001] reduced risk of mild vs. moderate and severe NAFLD in young adult women. Our findings suggest that the serum iron:ferritin ratio more accurately predicts body composition and reduced risk of severe fatty liver progression in young adult women compared to middle-aged women.

## 1. Introduction

Nonalcoholic fatty liver disease (NAFLD) is the most common cause of chronic liver disease worldwide [1]. However, mechanisms underlying the etiology and progression of NAFLD remain unclear. NAFLD can progress from fat accumulation in the liver (steatosis) to nonalcoholic steatohepatitis (NASH) or from NASH to fibrosis or cirrhosis. Recently, NAFLD is considered to be the hepatic manifestation of metabolic syndrome (MetS) because NAFLD is strongly associated with obesity and the clustering of metabolic risk factors, including dyslipidemia and insulin resistance (IR) [2,3]. Emerging evidence suggests that iron contributes to NAFLD progression [3]. Although the role of hepatic iron in the progression of NASH, fibrosis, or cirrhosis remains inconclusive [4], dysmetabolic iron overload syndrome (DIOS) is commonly associated with obesity or obesity-related comorbidities, such as NAFLD and MetS [5]. Iron has been reported to serve as “the first hit” to promote liver steatosis through lipid interference [6] or glucose metabolism [7]. IR or iron-mediated lipid peroxidation and oxidative stress can trigger necrotic inflammation leading to the progression of steatosis to NASH and, subsequently, to cirrhosis [8]. Furthermore, elevated hemoglobin and serum ferritin levels are independent risk factors for NAFLD [9,10].

Iron biomarkers have been reported to predict central obesity; the effects of this relationship on NAFLD progression remain unclear [11,12]. Altered body composition is intimately associated with NAFLD and adverse health outcomes [13]. A human study showed a positive correlation between elevated serum ferritin levels and visceral or trunk fat mass [12]. Animal studies have reported that diet-induced obese animals had iron retention in the liver or adipose tissues and that tissue iron overload is associated tissue inflammation [14,15]. Aigner et al. investigated iron regulatory proteins in patients who had NAFLD, both with (*n* = 32) and without iron overload (*n* = 29) and observed that hepatic iron accumulation may result from ineffective iron sensing and iron export because of altered hepcidin-ferroportin-hemojuvelin expression [16]. Obesity-related inflammation is known to induce hepcidin synthesis, and elevated serum hepcidin levels are associated with hypoferremia, elevated serum ferritin levels, and tissue iron overload [17]. Tissue iron retention may trigger chronic tissue inflammation and the wound healing process, which may promote hepatic NASH or fibrosis. Conversely, decreased muscle mass may affect the glucose utilization of the body, and muscle IR may further enhance liver injury [13,18]. 

Excess intake of energy or high consumption of fructose, trans-fatty acids, and saturated fat are associated with NAFLD [19]. Weight loss of 5% due to hypocaloric diet or the combination of a hypocaloric diet and moderate exercise can reduce hepatic steatosis and improve NASH [20]. The Mediterranean has health benefit and may prevent obesity and NAFLD [21]. However, no consensus is available on the most effective diet for NAFLD treatment [19]. Currently, nutritional risk factors in NAFLD patients with dysregulated iron metabolism are also not known. This study investigated the association between serum iron biomarkers and fatty liver severity in 117 adult women (control: *n* = 19 and NAFLD: *n* = 98). The specific aims were as follows: (1) to assess the age-specific association between serum iron biomarkers and body composition; and (2) to identify the iron-specific dietary pattern associated with fatty liver severity.

## 2. Materials and Methods

### 2.1. Study Design

This cross-sectional study was performed at the Division of Gastroenterology and Hepatobiliary Disease, Department of Internal Medicine in the Taipei Medical University Hospital between July 2015 and June 2016. This study was conducted in Taiwan and all subjects were Han Chinese. This study was approved by the Institutional Ethical Review Committee of the Taipei Medical University (TMU-JIRB 201502018). Written informed consent was obtained from all participants. 

### 2.2. Data Collection

Participants were excluded if they had (i) a history of hepatitis virus infection (e.g., hepatitis virus A, B, or C); (ii) a history of cholecystectomy and drug-induced hepatitis; (iii) excessive alcohol consumption, defined by an alcohol intake of >20 g/week for women; and (iv) chronic diseases (e.g., hepatocellular carcinoma, nephritis, cancer, and autoimmune disease); or were (v) pregnant, breastfeeding, and used hormone replacement therapy. In total, 117 women were recruited for analysis. 

### 2.3. Definition of Diseases

Abdominal ultrasound was performed for all participants by experienced gastroenterologists to diagnose fatty liver severity. The severity was independently graded by two gastroenterologists as normal (grade 0), mild (grade 1), moderate (grade 2), and severe (grade 3) [22]. Nineteen women were classified as having normal livers and 98 women were classified as having fatty livers (mild: *n* = 67, moderate: *n* = 25, and severe: *n* = 6). MetS was defined as patients with at least three of the following criteria based on the modified National Cholesterol Education Program Adult Treatment Panel III for Asia Pacific: (i) waist circumference ≥80 cm; (ii) systolic blood pressure ≥130 mmHg or diastolic blood pressure ≥85 mmHg; (iii) fasting blood glucose ≥110 mg/dL; (iv) high-density lipoprotein cholesterol (HDL-C) <50 mg/dL; and (v) fasting triglycerides (TGs) ≥150 mg/dL [23]. Diabetes mellitus was defined as hemoglobin A1c (HbA1c) >6.5% or self-reported. Dyslipidemia was defined as patients with at least one of the following criteria: (i) TGs ≥200 mg/dL; (ii) total cholesterol ≥240 mg/dL; (iii) HDL-C <35 mg/dL; (iv) low-density lipoprotein cholesterol (LDL-C) ≥160 mg/dL; (v) total cholesterol:HDL-C ≥5; and (vi) use of lipid-lowering drugs [24]. Anemia was defined as Hb <12 g/dL. Furthermore, iron deficiency was noted if both iron indicators showed abnormal values: serum ferritin (SF) ˂12 ng/mL and percentage of transferrin saturation (%TS) <15% [25]. Iron deficiency anemia (IDA) was defined as SF ˂12 ng/mL, %TS <15%, and Hb <12 g/dL. Iron overload was defined as SF >200 ng/mL [26]. 

### 2.4. Evaluation of Body Composition

The body mass index (BMI) was calculated as mass (kg)/[height (m)]^2^. Overweight and obesity were defined based on the criteria of the World Health Organization for Asia, which defines overweight as BMI ≥24 kg/m^2^ and obesity as BMI ≥27 kg/m^2^ [27]. Central obesity was defined as waist circumference ≥80 cm. The body composition was measured by bioelectrical impedance analysis (BIA) by using a direct segmental multifrequency BIA meter (X-SCAN Plus-II analyzer; Jawon, Korea). Body compositions were divided by body weight and expressed as percent skeletal muscle, percent body fat, percent visceral fat, and percent subcutaneous fat.

### 2.5. Blood Biochemistry Examination

Fasting blood samples were collected from overnight fasting participants, and serum and plasma were stored at −80 °C until analysis. Heparinized whole blood was collected for Hb measurement. Serum iron and total iron-binding capacity (TIBC) were measured using a ferrozine-based colorimetric method. %TS was determined as (serum iron/TIBC) × 100. Serum ferritin was measured by electrochemiluminescence immunoassay and was quantitated with a Roche Modular P800 analyzer (Mannheim, Germany). Furthermore, serum aspartate aminotransferase (AST) and alanine aminotransferase (ALT) were measured using a colorimetric method. Serum-free Hb (free Hb; Immunology Consultants Laboratory, Inc., Portland, OR, USA), serum hepcidin (DRG International Inc; Springfield, NJ, USA), and serum Nε-(carboxymethyl)lysine (CML; Cell Biolabs, San Diego, CA, USA) were analyzed by enzyme-linked immunosorbent assay, according to the manufacturer’s instructions. 

### 2.6. Dietary Assessment

A self-reported food frequency questionnaire (FFQ) was used to determine the dietary pattern of the participants. Dietary data were assessed using a modified Chinese version of the FFQ for the Taiwanese population [28]. The modified questionnaire comprises three components: the intake frequency of 66 food items, frequency of eating away from home, and cooking methods used. The food groups were as follows: bread and pastries, noodles, rice, whole grains, root starch, brightly-colored vegetables, seaweed, white and light green vegetables, dark green vegetables, fruits, desserts, western dishes, fried desserts, organs, processed meat, animal fats, dairy products, coffee, sugary beverages, beef and lamb, chicken and pork, duck and goose, seafood, soy products, eggs, homemade food, eating away from home, deep-fried food, grilled or barbecued food, stir-fried food, stewed food, and steamed/boiled/raw food. The FFQ was divided into eight levels: (1) 0–1 times/week; (2) 2–3 times/week; (3) 4–5 times/week; (4) 6–7 times/week; (5) 8–10 times/week; (6) 11–13 times/week; (7) 14–16 times/week; and (8) ≥17 times/weeks. No portion sizes were available.

### 2.7. Statistical Analyses

Statistical analyses were conducted using SPSS 19 (IBM Corp., Armonk, NY, USA) and SAS Version 9.4 (SAS Institute Inc., Cary, NC, USA). Categorical and continuous variables are presented as numbers (percentages) and mean ± standard deviation, respectively. Middle-aged women were defined as those aged ≥45 y, and young adult women were those younger than 45 y. For comparing baseline characteristics between young adult and middle-aged women, the Mann–Whitney U and chi-squared tests were used for analyzing continuous and categorical variables, respectively. Multivariate linear regression was performed to evaluate the associations between the dependent variables (percent visceral fat or percent muscle mass) and potential variables, including iron biomarkers and age. Odds ratio (OR) and 95% confidence intervals (CIs) were calculated to estimate fatty liver progression. The trend test was performed using simple linear regression after age adjustment. *p* < 0.05 was considered statistically significant.

**Figure 1:**
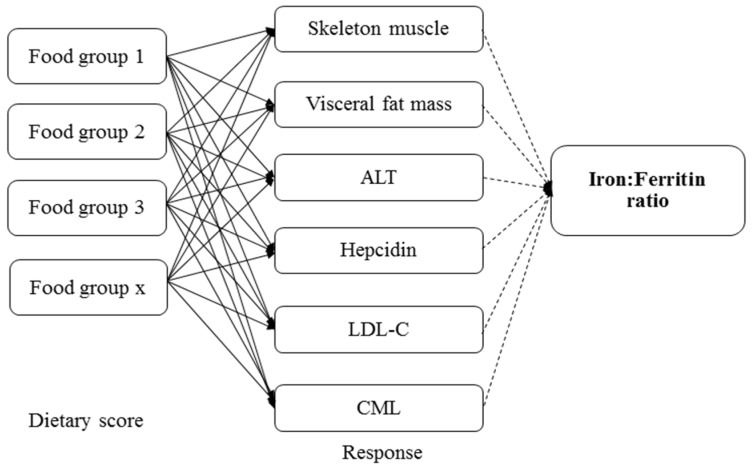
Directed acyclic graph of reduced rank regression. ALT, alanine aminotransferase; LDL-C, low-density lipoprotein cholesterol; CML, Nε-(carboxymethyl)lysine.

The iron:ferritin-specific dietary patterns were determined using reduced rank regression (RRR). This technique was described in detail by Hoffmann et al. [29] and has been applied in studies of dietary patterns and disease prediction [30,31,32]. In RRR, the food groups and biomarkers were considered as predictor and response variables, respectively. In this analysis, we used 32 food groups as the predictor variables. Five food groups with the highest factor loadings (≥0.20) and five with the lowest factor loadings (≤0.20) were used to describe the serum iron:ferritin dietary pattern. The response variables were as follows: percent skeleton muscle mass, percent visceral fat mass, serum ALT, hepcidin, LDL-C, and CML. The response variables were selected based on a significant correlation between serum iron:ferritin ratio and response variables by partial correlation after adjustment for age (*r* = 0.312 to −0.422; all *p* < 0.05). The directed acyclic graph in Figure 1 explains the RRR conceptual framework.

## 3. Results

### 3.1. Participant Characteristics

In total, 117 adult women (19 controls and 98 patients with NAFLD) were recruited for analyses. The mean age was 43.02 ± 13.32 y (controls: 38.99 ± 14.35 y and patients with NAFLD: 43.80 ± 13.04 y). A total of 33 middle-aged women were self-reported to be post-menopause (63.5%) and all of the young adult women had not entered post-menopausal stage. The overall prevalence rates of dyslipidemia, MetS, anemia, and iron overload were 37.2%, 22.3%, 13.3%, and 9.7%, respectively. We then stratified the participants by age. Table 1 shows that middle-aged women (≥45 y) were heavier and had higher prevalence of dyslipidemia, MetS, and severe fatty liver than did young adult women (<45 y; all *p* < 0.05; Table 1). Contrastingly, young adult women had a higher percent skeletal muscle mass and lower percent visceral fat mass and body fat mass than did middle-aged women (all *p* = 0.001; Table 1). Furthermore, young adult women had higher levels of serum iron, but lower levels of ferritin, hepcidin, HbA1c, and free Hb than did middle-aged women (all *p* < 0.001; Table 1). 

### 3.2. Associations among Serum Iron:Ferritin Ratio, Body Composition, and Fatty Liver Severity

**Figure 2:**
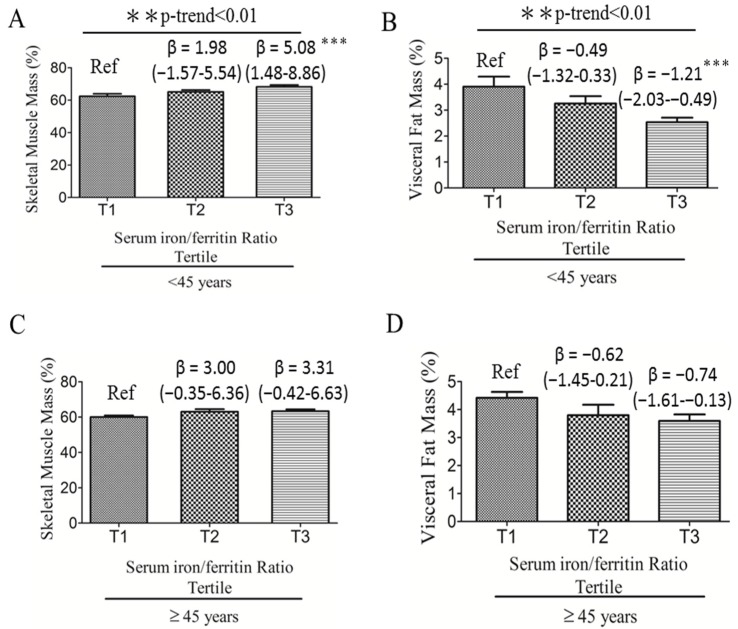
Age-adjusted linear regression analyses of serum iron:ferritin ratio and body compositions according to the tertile group of serum iron:ferritin ratio in young adult (*n* = 65) and middle-aged (*n* = 52) women. (**A**) Serum iron:ferritin ratio to predict skeletal muscle mass in women <45 years; (**B**) Serum iron:ferritin ratio to predict visceral fat mass in women <45 years; (**C**) Serum iron:ferritin ratio to predict skeletal muscle mass in women ≥45 years; (**D**) Serum iron:ferritin ratio to predict visceral fat mass in women ≥45 years. ** *p* < 0.01, and *** *p* < 0.001 indicate statistical significance.

We investigated the association between serum iron:ferritin ratio and body composition in women. Figure 2 shows that young adult women with the highest serum iron:ferritin ratio tertile (Tertile 3; T3) had a 5.08-unit increased percent muscle mass [β = 5.08 (1.48–8.68), *p* < 0.001; Figure 2A] and a 1.21-unit decreased percent visceral fat mass [β = −1.21 (−2.03 to −0.39), *p* < 0.001; Figure 2B] compared with those with the lowest serum iron:ferritin ratio (T1) (Reference; Ref). No significant associations were observed between body compositions and the iron:ferritin ratio in middle-aged women. 

Age-adjusted multivariate logistic regression analysis revealed that the serum iron:ferritin ratio in young adult women was associated with a 47% reduced [OR = 0.53, 95% CI: 0.30–0.95, *p* < 0.05] risk of progression from mild to moderate and severe fatty liver (Model 1, Table 2). However, no such relationship was observed in middle-aged women [OR = 1.01, 95% CI: 0.91–1.11; Model 1, Table 2].

### 3.3. Iron:Ferritin-Specific Dietary Pattern Is Associated with Reduced Risk of Fatty Liver Progression in Young Adult Women

We investigated the dietary pattern associated with the iron:ferritin ratio and determined whether this pattern predicted fatty liver severity. Table 3 shows items that such as beef and lamb, dairy products, fruits, whole grain, and eggs, were positively correlated with dietary pattern scores (factor loadings ≥0.20). Contrastingly, rice, noodles, bread and pastries, and stir- and deep-fried foods were negatively correlated with dietary pattern scores (factor loadings ≤0.20).

**Figure 3:**
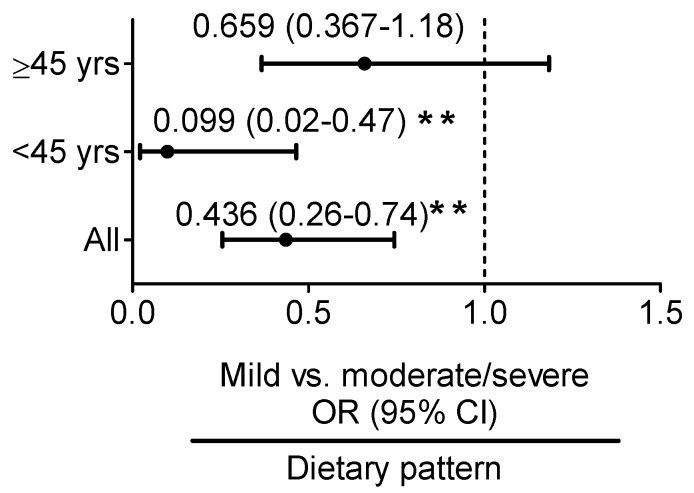
Age-adjusted odds ratio (OR) and 95% confidence interval (CI) for predicting mild vs. moderate and severe fatty liver according to the serum iron:ferritin-specific dietary pattern score determined using reduced rank regression. ** *p* < 0.01 indicate statistical significance.

Age-adjusted multivariate logistic regression analysis revealed that in young adult women, the iron:ferritin-specific dietary pattern was associated with a 90% reduced [OR = 0.10, 95% CI: 0.02–0.47, *p* < 0.01] risk of progression from mild to moderate and severe fatty liver (Figure 3). No such relationship was observed in middle-aged women.

## 4. Discussion

Our study revealed that the serum iron:ferritin ratio was associated with a healthy body composition, indicated as increased muscle mass and decreased visceral fat mass, and a reduced risk of moderate and severe fatty liver progression in young adult women, but not in middle-aged women. The study findings suggest that age or age-related factors (e.g., obesity) affect the body iron status and influence fatty liver progression in women. The serum iron:ferritin-specific dietary pattern (high intake of beef and lamb, dairy products, eggs, fruits, and whole grains and low intake of refined carbohydrates and stir- and deep-fried foods) can supply adequate iron to young adult women. This pattern was also associated with a 90% reduced risk of mild vs. moderate and severe fatty liver progression. Hence, it is likely that the serum iron:ferritin ratio reflects an adequate supply of iron to maintain a healthy body composition and, consequently, protect the liver against iron overload-related liver injury.

Approximately 5–10% of the body’s iron is stored in myoglobin. Iron is an essential nutrient for muscle growth and performance. For example, muscles require iron-containing proteins for oxygen transportation and oxidation. An early study reported that serum iron exerts lipolytic effects in isolated adipocytes [33]. Elevated serum ferritin, considered as an acute-phase reactant, is associated with visceral fat mass and NAFLD, suggesting that serum iron or ferritin has direct or indirect effects on adiposity and liver function. However, correlations between the serum iron:ferritin ratio and body composition may be disrupted by obesity-related factors in middle-aged women. Our study revealed that middle-aged women were heavier and had higher levels of serum hepcidin and ferritin and lower levels of serum iron than did young adult women. Increased hepcidin is well-established to gradually cause hypoferremia and tissue iron overload. Moreover, decreased serum iron levels may affect muscle growth in middle-aged women. Hence, elevated serum hepcidin and ferritin are likely to reflect a degree of chronic inflammation and tissue iron retention in middle-aged women. Overall, the serum iron:ferritin ratio may not only reflect the bioavailability of iron for the body, but also a healthy body composition. Disruptions in tissue iron sequestration because of elevated serum hepcidin may result in an unhealthy body composition and fatty liver progression.

Our study showed that the high consumption of beef and lamb, dairy products, eggs, fruits, and whole grains and low consumption of refined carbohydrates (rice, noodles, and bread and pastries) as well as deep- and stir-fried foods protect young adult women against fatty liver progression. These food groups explained approximately 90% of the variation in the dietary pattern scores. Beef and lamb consumption explained approximately 17.8% of the variation in the serum iron:ferritin dietary pattern scores. Consistent with our result, Hodgson et al. reported an association between lean red meat and increased serum iron levels, but not serum ferritin levels [34]. Doyle et al. examined 1268 elderly patients and reported that a varied diet (meat, poultry and fish, and vegetables and fruits, with a moderate alcohol consumption) was positively correlated with serum iron status in elderly patients [35]. Approximately 40% of iron in red meat is heme iron, which contains ferrous iron and has a higher absorption rate than does non-heme iron [36]. However, red meat is a risk factor for NAFLD [37]. In Taiwan, pork is the most consumed red meat (31.3 kg per capita per year) compared with beef and lamb (3.9 and 2.9 kg per capita per year, respectively) [38]. Adequate meat consumption is important for increasing muscle mass. A study suggested that 25–30 g of protein per meal can stimulate muscle protein synthesis [39]. Hence, it seems reasonable to advise young adult women to moderately consume beef and lamb to improve their iron and muscle mass statuses. In the present study, eggs accounted for approximately 9.1% of the variation in the serum iron:ferritin dietary pattern scores. Eggs are rich in protein and cholesterol. Mokhtari et al. reported that the consumption of more than four eggs per week had no effect on NAFLD [40]. Our study also reported that a high consumption of whole grains and fruits (accounting for 11.5% of the variation in the dietary pattern scores) and low consumption of deep- and stir-fried foods and refined carbohydrates (accounting for 44.4% of the variation in the dietary pattern scores) protect against fatty liver progression. Whole grains are rich in ferric iron. Fruits contain vitamin C, which can facilitate ferric iron absorption in the small intestine. Georgoulis et al. showed that switching from refined carbohydrates to whole grains can reduce abdominal fat and prevent NAFLD [41]. Dry-heat cooking methods, such as deep-frying, may increase the levels of exogenous advanced glycation end products (AGEs) by the Maillard reaction during dry-heat processing. Recent evidence suggested that serum CML, a major immunogen of AGEs, was accumulated in the liver and positively correlated with NAFLD severity [42].

Our resultsuggested that middle-aged women were more likely to have central obesity (30% vs. 59%, *p* = 0.002) and obesity-related comorbidities, such as MetS (13% vs. 35%, *p* = 0.006), dyslipidemia (28% vs. 49%, *p* = 0.02), and moderate and severe FLD (15% vs. 40%, *p* = 0.006), than were young adult women. Despite low serum iron levels in middle-aged women (99.7 vs. 92.1 μg/L, *p* < 0.001), the prevalence rates of anemia (14% vs. 12%), iron deficiency (8% vs. 6%, *p* = 0.73), and IDA (8% vs. 6%, *p* = 0.73) did not vary between young adult and middle-aged women in our study. Normally, reproductive-age women are more likely to have iron deficiency than are middle-aged women because of menstrual blood loss or increased iron demand during pregnancy and lactation. Our data suggest that, despite the low prevalence rate of iron deficiency, middle-aged women are more likely to develop altered iron metabolism because of obesity-related comorbidities and elevated serum hepcidin. Altered iron metabolism may affect the body composition, which consequently, may accelerate NAFLD progression.

The study has several limitations. First, the relatively small sample size and cross-sectional design limited the study findings. The relative low number of moderate (*n* = 25) and severe FLD (*n* = 6) among women is another limitation. The prevalence of hepatitis virus infection in Taiwan is high. Therefore, the number of moderate and sever FLD are low after the exclusion of patients with hepatitis virus infection (e.g., hepatitis virus A, B, or C). Second, NAFLD was diagnosed based on abdominal ultrasonography and not liver biopsy. Abdominal ultrasonography is commonly used for diagnosing NAFLD in Taiwan because it offers relatively low cost, non-invasiveness, and fewer side effects than liver biopsy. Third, the body composition was evaluated using a bioelectrical impedance analysis device, and not dual-energy X-ray absorptiometry, because of budget constraints. Fourth, we used RRR to generate iron:ferritin-specific dietary pattern scores; this method requires in-depth knowledge of the diet–disease relationship to select intermediate variables. Our study selected six response variables as the intermediate variables, namely skeletal muscle mass, visceral fat mass, ALT, hepcidin, LDL-C, and CML. These were based on the literature on the association between iron and NAFLD, as well as the correlation between the serum iron:ferritin ratio and response variables. However, selecting response variables can be subjective and personal and may result in different dietary patterns in different age groups or studies. For example, the intermediate variables may be influenced by age, sex, or disease status. In our study, the association between the serum iron:ferritin ratio and response variables varied between young and middle-aged women, suggesting that age has direct effects on the intermediate variables and influences the diet–disease relationship. Future studies are required to validate our findings in a large cohort study. Moreover, it is important to identify dietary factors that may contribute to altered iron metabolism in middle-aged women because this population has a high risk of anemia of inflammation and severe fatty liver injury.

## 5. Conclusions

Our findings suggest that the serum iron:ferritin ratio predicts a healthy body composition and reduced risk of severe fatty liver in young adult women. The increased consumption of beef and lamb, dairy products, fruits, whole grains, and eggs, and decreased consumption of refined carbohydrates (rice, noodles, bread and pastries), as well as deep- and stir-fried foods may supply adequate iron to young adult women to maintain a healthy body composition and prevent fatty liver progression. Additional studies are required to determine dietary risk factors for iron metabolism alterations and fatty liver progression in middle-aged women. 

## Figures and Tables

**Table 1:** Clinical characteristics of women stratified by age.

Characteristics	<45 Y (*n* = 65)	≥45 Y (*n* = 52)	*p* Value *
Age (y)	32.85 ± 7.30	55.73 ± 6.30	<0.001
BMI (kg/m^2^)	23.27 ± 6.49	23.60 ± 3.97	0.040
Waist circumference (cm)	79.57 ± 15.09	80.63 ± 9.3	0.017
Overweight and obese (*n*, %)	17 (26.2%)	21 (40.4%)	0.102
Central obesity (*n*, %)	19 (29.7%)	30 (58.7%)	0.002
Diabetes mellitus (*n*, %)	2 (3.1%)	5 (9.6%)	0.318
Dyslipidemia (*n*, %)	18 (28.1%)	24 (49.0%)	0.023
MetS (*n*, %)	8 (12.7%)	17 (34.7%)	0.006
NAFLD stage (*n*, %)			0.006
Control	14 (21.5%)	5 (9.6%)	
Mild	41 (63.1%)	26 (50.0%)	
Moderate/severe	10 (15.4%)	21 (40.4%)	
Self-reported post-menopause	0 (0%)	33 (63.5%)	<0.001
Anemia (*n*, %)	9 (13.8%)	6 (11.5%)	0.778
Iron deficiency (*n*, %)	5 (7.7%)	3 (5.7%)	0.728
Iron deficiency anemia (*n*, %)	5 (7.7%)	3 (5.7%)	0.728
Iron overload (*n*, %)	6 (9.2%)	5 (9.6%)	0.883
**Inflammation markers**			
AST (U/L)	21.94 ± 9.69	28.39 ± 13.47	<0.001
ALT (U/L)	22.52 ± 18.21	32.14 ± 25.42	<0.001
**Body compositions**			
Body fat mass (%)	29.02 ± 6.37	32.31 ± 5.07	0.001
Skeletal muscle mass (%)	65.27 ± 6.30	62.00 ± 5.02	0.001
Visceral fat (%)	3.22 ± 1.43	3.97 ± 1.25	<0.001
Subcutaneous fat (%)	25.92 ± 5.02	28.34 ± 3.85	0.002
**Serum iron biomarkers**			
Iron (µg/dL)	99.66 ± 42.35	92.12 ± 35.19	<0.001
TS (%)	26.67 ± 12.65	25.91 ± 10.88	0.993
Ferritin (ng/mL)	54.30 ± 72.72	98.81 ± 83.97	<0.001
Iron:ferritin ratio	4.41 ± 3.39	2.12 ± 2.59	<0.001
Hepcidin (ng/mL)	79.90 ± 107.59	139.17 ± 121.39	0.027
Hb (g/dL)	14.03 ± 3.01	13.30 ± 2.13	0.402
HbA1c (%)	5.50 ± 0.66	6.16 ± 1.44	<0.001
Free Hb (µg/mL)	133.34 ± 55.73	164.95 ± 56.03	0.016
CML (µg/mL)	258.14 ± 149.01	246.81 ± 145.19	0.720

Continuous data are presented as mean ± standard deviation; categorical data are presented as numbers (percentages). * *p* value was analyzed using the Mann–Whitney test for continuous variables and chi-squared test for categorical variables. Abbreviations: BMI = Body mass index, MetS = metabolic syndrome, AST = aspartate aminotransferase, ALT = alanine aminotransferase, Hb = hemoglobin, Free Hb = free hemoglobin, CML = Nε-(carboxymethyl)lysine.

**Table 2:** Age-adjusted odds ratios (OR) and 95% confidence intervals (CIs) for fatty liver severity.

Iron:Ferritin Ratio	Control vs. Mild Fatty Liver		Mild vs. Moderate/Severe Fatty Liver	
	OR (95% CI)	*p* Value	OR (95% CI)	*p* Value
All				
Univariate	0.923 (0.795–1.072)	0.295	0.734 (0.576–0.935)	0.012
model 1	0.929 (0.791–1.092)	0.373	0.799 (0.626–1.021)	0.073
<45 years				
Univariate	0.931 (0.783–1.107)	0.419	0.508 (0.281–0.917)	0.025
model 1	0.932 (0.783–1.110)	0.430	0.531 (0.298–0.946)	0.032
≥45 years				
Univariate	1.017 (0.621–1.666)	0.947	0.953 (0.752–1.208)	0.691
model 1	0.933 (0.551–1.579)	0.796	1.006 (0.908–1.114)	0.912

**Table 3:** Percentage of food variations explained by the first dietary pattern scores and factor loadings of all 32 food groups derived from reduced rank regression in all participants (*n* = 117).

Food Groups	Explained Variations (%)	Factor Loadings *
Beef and lamb	17.79	0.38
Dairy products	9.07	0.27
Fruits	8.03	0.26
Whole grains	6.74	0.24
Eggs	4.71	0.20
Rice	12.54	−0.32
Noodles	9.20	−0.28
Bread and pastries	8.26	−0.26
Stir-fried food	7.87	−0.26
Deep-fried food	6.50	−0.23
Organs	4.99	−0.20
Steamed/boiled/raw food	3.94	0.18
Dark green vegetables	3.78	0.18
Western dishes	2.80	0.15
White and light green vegetables	2.09	0.13
Orange, red, and purple vegetables	2.07	0.13
Coffee	1.63	−0.12
Stew food	1.57	−0.11
Seafood	1.30	0.10
Fried desserts	1.03	0.09
Homemade food	0.80	−0.08
Animal fats	0.75	0.08
Grilled or barbecued food	0.74	0.08
Root starch	0.54	−0.07
Seaweed	0.48	−0.06
Desserts	0.40	0.06
Eat away from home	0.30	−0.05
Duck and goose	0.28	−0.05
Processed meats	0.17	0.04
Soy products	0.02	0.01
Chicken and pork	0.01	0.01
Sugar beverages	0.00	0.00

* Factor loadings showed correlations between food groups and the first dietary pattern score.

## Abbreviations

ALT, alanine aminotransferase; AST, aspartate aminotransferase; BMI, body mass index; CI, confidence interval; CML, Nε-(carboxymethyl)lysine; DIOS, dysmetabolic iron overload syndrome; FFQ, frequency questionnaire; Free Hb, free hemoglobin; HDL-C, high density lipoprotein cholesterol; Hb, hemoglobin; IDA, iron deficiency anemia; IR, insulin resistance; LDL-C, low density lipoprotein cholesterol; MetS, metabolic syndrome; NAFLD, nonalcoholic fatty liver disease; NASH, nonalcoholic steatohepatitis; OR, odds ratio; RRR, reduced rank regression; SD, standard deviation; SF, serum ferritin; TGs, triglycerides; %TS, percentage transferrin saturation; TIBC, total iron-binding capacity.
